# Molecular Changes in the Adipose Tissue Induced by Rheumatoid Arthritis: Effects of Disease-Modifying Anti-Rheumatic Drugs

**DOI:** 10.3389/fimmu.2021.744022

**Published:** 2021-10-13

**Authors:** Iván Arias de la Rosa, Alejandro Escudero-Contreras, Miriam Ruiz-Ponce, Cristóbal Román-Rodríguez, Carlos Pérez-Sánchez, María del Carmen Ábalos-Aguilera, Rafaela Ortega-Castro, Juan Alcaide, Mora Murri, Pilar Font, Jerusalem Calvo-Gutiérrez, Maria Luque-Tevar, Alejandra Maria Patiño-Trives, Rocío Guzmán-Ruiz, Maria del Mar Malagón, Francisco José Tinahones, Eduardo Collantes-Estévez, Chary López-Pedrera, Nuria Barbarroja

**Affiliations:** ^1^ Rheumatology Service/Deparment of Medicine and Surgical Sciences, Maimonides Institute for Research in Biomedicine of Cordoba (IMIBIC), University of Cordoba, Reina Sofia Hospital, Cordoba, Spain; ^2^ Biomedical Research Institute (IBIMA), Service of Endocrinology and Nutrition, Malaga Hospital Complex, Virgen de la Victoria, Malaga, Spain; ^3^ Centros de Investigación Biomédica en Red (CIBER) Fisiopatología de la Obesidad y Nutrición (CIBEROBN), Instituto de Salud Carlos III, Madrid, Spain; ^4^ Department of Cell Biology, Physiology and Immunology, Maimonides Institute for Research in Biomedicine of Cordoba (IMIBIC), Reina Sofia University Hospital, University of Cordoba, Cordoba, Spain

**Keywords:** rheumatoid arthritis, obesity, inflammation, adipose tissue, cDMARDs, leflunomide, hydroxychloroquine, methotrexate

## Abstract

Disease severity, progression and response to therapy might be worse in obese rheumatoid arthritis (RA) patients, but paradoxically, obesity also might protect from radiographic joint damage. Thus, the intricate relationship between obesity and RA needs urgent clarification. The aim of this study was to assess the influence of obesity on the onset and development of RA and to determine whether arthritis could modify the adipose tissue biology and whether conventional Disease Modifying Anti-Rheumatic Drugs (cDMARDs) can modulate these alterations. Two strategies were followed: (1) clinical profiling of two cohorts of RA: non-obese and obese patients; and (2) mechanistic studies carried out in both a collagen-induced arthritis (CIA) in an obese mouse model and 3T3-L1 adipocytes treated with cDMARDs (leflunomide, methotrexate, and hydroxychloroquine). In our cohort of RA patients with low-moderate disease activity, the presence of obesity was not related to a higher activity of the disease; actually, disease activity score 28-erythrocyte sedimentation rate (DAS28-ESR) was reduced in the obese RA patients. However, the induction of arthritis promoted transcriptomic changes in the adipose tissue under obesity condition in the obese CIA model. Treatment with hydroxychloroquine reduced weight and insulin resistance, accompanied by beneficial metabolic effects in the adipose tissue. These molecular changes in adipose tissue were also observed after methotrexate administration. In sum, arthritis might affect directly the inflammatory burden and metabolic alterations associated with obesity in adipose tissue. Clinicians should be cautious measuring the activity of the disease in obesity and managing the best therapeutic options for the metabolic comorbidities of these patients, where the combination of hydroxychloroquine and methotrexate should be considered to improve adipose tissue dysfunction in obese RA.

## Introduction

Rheumatoid arthritis (RA) is a chronic inflammatory autoimmune disease that comprises joint inflammation and bone destruction which might cause disability. RA is closely associated with diverse comorbidities, including cardiovascular disease, which is reported to be the primary cause of death in RA (1). In this sense, environmental risk factors include infections, smoking, periodontitis, hormonal and dietary factors, physical inactivity, type 2 diabetes mellitus, and obesity. RA and obesity share mechanisms of inflammation, which make plausible a strong link between these two diseases. About two-thirds of people with RA are overweight or obese. In fact, RA since early stages is associated with altered body composition, characterized by reduced lean mass and often increased fat mass (2), which might accelerate morbidity and mortality (3). Furthermore, obesity maintains a systemic low-grade inflammatory environment provoked by dysfunctional adipose tissue that releases high levels of inflammatory cytokines (4), which can amplify autoimmune disorders and their associated comorbidities, being the reason why it might be considered a risk factor for RA. Adipose tissue is now recognized as an active endocrine organ, constituted not only by adipocytes but also by a number of immune cells such as macrophages, neutrophils, and T and B cells (5, 6). The adipocyte expansion caused by positive energy balance leads to adipocyte hypoxia, apoptosis, and cell stress, ultimately resulting in the expression of chemoattractant molecules and increased infiltration of inflammatory cells (6). The obese adipose tissue is also characterized by a markedly deregulated production of adipose tissue-derived factors, i.e., adipokines, a growing family of low molecular weight, biologically active proteins with pleiotropic functions (7). Adipokines are crucial players not only in energy metabolism but also in inflammation and immunity, most of them being increased in obesity and contributing to the associated “low-grade inflammatory state” (8). This demonstrates that in addition of the role of adipose tissue as an energy storage under conditions of caloric surplus, it is an active participant in regulating physiologic and pathologic processes such as immunity and inflammation. It has been suggested that the increased inflammation associated with obesity contributes to the activity of RA and comorbidities alike (cardiovascular disease, type 2 diabetes, chronic pulmonary disease), decreasing the quality of life of RA patients as well as the remission rates of the disease (9–11). However, paradoxically, it has been shown that obesity protects from radiographic joint damage (12). While several studies have examined the potential influence of obesity on the development of RA, the results have been inconsistent (13, 14). The aim of this study was to assess the influence of obesity on the onset and development of RA, and to determine whether arthritis could modify the adipose tissue biology in an obesity context and how cDMARDs could modulate these metabolic alterations.

## Materials and Methods

### Patients

This is a cross-sectional study including 290 human participants. One hundred and fifty RA patients (100 lean and 50 obese) and 100 lean and 40 obese, age- and gender-matched controls were included in this study. RA patients fulfilled at least four 1987 American College of Rheumatology (ACR) and achieved a total score of 6 or greater according to 2010 criteria (15, 16). All RA patients were tested for the presence of anticyclic citrullinated peptides (anti-CCPs) and rheumatoid factor (RF). Disease activity score 28 (DAS28) index was determined following the guidelines of the American College of Rheumatology indications. None of the lean and obese controls had a history of other autoimmune diseases, atherothrombosis, and thrombosis. The participants enrolled were Caucasian and recruited at both the department of rheumatology in the Reina Sofia Hospital (Cordoba, Spain) and the department of endocrinology in the Virgen de la Victoria Hospital (Malaga, Spain), after approval from the ethics committee of the Reina Sofia Hospital (code PI17/01316). Metabolic features [lipid profile, body mass index (BMI), glucose, and insulin], disease activity, and features of disease-modifying antirheumatic drug (DMARD) and glucocorticoid therapy were recorded (**Table 1**). Obesity was defined according to BMI >30. Disease activity score 28 (DAS28) variables comprised the erythrocyte sedimentation rate (ESR), the swollen joint count (in 28 joints), the tender joint count (in 28 joints), and the patient’s assessment of disease activity [measured on a 0–100 mm visual analogue scale (VAS)]. Laboratory tests were performed on blood samples obtained from patients who were fasted during 8 h. The method used to measure insulin resistance was homeostatic model assessment-insulin resistance (HOMA-IR) index: [insulinemia (mU/L) × glycemia (mg/dl)]/405. HOMA-IR values >2.5 indicated insulin resistance (17, 18).

**Table 1:** Clinical details of the four cohorts included in the study: RA-non-obese patients, RA-obese patients, healthy donors, and obese non-RA subjects.

	Non-obese HD	Non-obese-RA	Obese-RA	Obese
**N**	50	100	40	100
**Female/Male (n/n)**	38/12	75/25	32/8	77/23
**Age (years)**	46.06 ± 11.02	57.36 ± 13.49	57.93 ± 11.90	53.89 ± 15.03
**Disease duration (years)**	NA	6.69 ± 6.10	7.01 ± 7.84	NA
**RF positive (%)**	NA	55.00	56.00	NA
**ACPAs positive (%)**	NA	66.00	53.00	NA
**Tender joints (n)**	NA	1.13 ± 2.09	0.32 ± 0.81^b^	NA
**Swollen joints (n)**	NA	3.56 ± 1.44	1.44 ± 2.01^b^	NA
**DAS28 (CRP)**	NA	2.94 ± 0.99	3.11 ± 0.78	NA
**DAS28 (ERS)**	NA	2.84 ± 1.18	2.39 ± 0.72^b^	NA
**VAS**	NA	39.01 ± 23.04	28.54 ± 17.03^b^	NA
**Smoker (%)**	20.00	25.00	11.00	11.00
**BMI (kg/m²)**	23.63 ± 3.4	25.28 ± 2.72	34.46 ± 4.44^a,b^	34.62 ± 1.47^a,b^
**Laboratory parameters**				
**Glucose (mg/dl)**	83.51 ± 9.26	93.06 ± 22.36	107.31 ± 47.06^a,b^	105.57 ± 25.08^a,b^
**Insulin (μUI/ml)**	5.85 ± 4.02	7.56 ± 3.48^a^	10.75 ± 5.78^a,b^	11.85 ± 6.85^a,b^
**HOMA-IR**	1.25 ± 0.89	1.85 ± 1.14^a^	3.01 ± 2.60^a,b^	3.10 ± 1.93^a,b^
**Cholesterol (mg/dl)**	197.58 ± 30.99	202.12 ± 36.08	204.96 ± 37.58	207.58 ± 45.74
**HDL-cholesterol (mg/dl)**	58.76 ± 15.28	56.51 ± 17.77	53.41 ± 12.62	50.79 ± 15.00
**LDL-cholesterol (mg/dl)**	120.87 ± 23.44	126.91 ± 30.90	126.17 ± 33.87	127.75 ± 37.26
**Triglycerides (mg/dl)**	87.56 ± 45.35	94.11 ± 42.14^a^	124.96 ± 50.65^a,b^	143.98 ± 71.21^a,b^
**ESR (mm/h)**	7.82 ± 4.49	13.89 ± 10.80^a^	13.02 ± 8.62^a^	ND
**CRP (mg/L)**	1.54 ± 2.13	4.88 ± 6.79^a^	9.36 ± 6.37^a,b^	6.61 ± 4.73^a^
**Treatments**				
**Corticosteroids (%)**	NA	37.00	40.00	NA
**Antimalarial (%)**	NA	41.00	50.00	NA
**NSAIDS (%)**	NA	68.00	70.00	NA
**Methotrexate (%)**	NA	60.00	50.00	NA
**Leflunomide (%)**	NA	32.00	25.00	NA

Values are mean ± SD. HD, Healthy donors; RA, Rheumatoid arthritis; RF, Rheumatoid Factor; ACPAs, anti-citrullinated protein antibodies; DAS, Disease Activity Score; VAS, visual analog scale; BMI, Body Mass Index; HDL, High density lipoprotein; LDL, Low density lipoprotein; ESR, Erythrocyte sedimentation rate; CRP, C-reactive protein; HOMA-IR, homeostatic model assessment–insulin resistance; NSAIDS, Non-steroidal anti-inflammatory drugs; NA, not applicable; ND, not determine. (a) Significant differences vs non-obese healthy donors (p<0.05); (b) Significant differences vs non-obese-RA patients (p<0.05).

### CIA Obese Mouse Model

All animal experiments complied with the ARRIVE guidelines and were carried out in accordance with the U.K. Animals (Scientific Procedures) Act, 1986 and associated guidelines, the National Institutes of Health guide for the care and use of laboratory animals (NIH Publications n° 8023, revised 1978). C57BL/6 mice, 4 weeks old, were housed in polycarbonate cages. They were fed 60 Kcal fat-derived calories and standard mouse chow and water *ad libitum*. Fifty-Five C57BL/6 mice (male, 4–5 weeks) were used in this study. Five mice were used as non-diseased controls (lean control), nine mice were used as lean collagen-induced arthritis (CIA), five mice were obese non-diseased (obese control), and 36 mice were used as obese CIA (nine obese CIA non-treated, nine CIA obese treated with leflunomide, nine CIA obese treated with methotrexate, and nine CIA obese treated with hydroxychloroquine). To induce CIA, mice were injected subcutaneously with collagen/CFA emulsion (100 μg/mouse); at day 21st, mice were boosted with collagen/ICFA (combination of collagen solution and incomplete Freud’s adjuvant) emulsion (100μμg/mouse). For the CIA obese group, the first collagen immunization was done when mice weighted 30 g. Treatment was administered by the time of the second immunization. Mice were treated with leflunomide (10 mg/kg daily), methotrexate (3 mg/kg three times per week) or hydroxychloroquine (60 mg/kg daily) for 15 days. The severity of arthritis was determined by three independent observers. Macroscopic signs of arthritis were scored three times weekly, where each paw received a score: 0 = no visible effects of arthritis; 1 = edema and/or erythema of 1 digit; 2 = edema and/or erythema of 2 digits; 3 = edema and/or erythema of more than 2 digits; 4 = severe arthritis of entire paw and digits. The arthritic index (AI) was calculated by addition of individual paw scores and recorded up to maximum of 16. Mice were weighted daily. After mice were sacrificed, gonadal adipose tissue and plasma were isolated and frozen at −80°C until analysis.

### Leptin and TNF-α Plasma Levels

Plasma levels of tumor necrosis factor-α (TNF-α) and leptin in mice were quantified through enzyme-linked immunosorbent assay (ELISA), following the manufacturer’s recommendation (Bionova, Diaclone). The assay employs the quantitative sandwich enzyme immunoassay technique. Antibodies specific for TNF-α and leptin were precoated onto a microplate. Samples and standards were pipetted into the wells, and any target molecule present was bound by the immobilized antibody. After removing any unbound substances, a biotin-conjugated antibody specific for TNF-α and leptin were added to the wells. After washing, avidin conjugated horseradish peroxidase (HRP) was added. Next, after removing any unbound substances, a substrate solution was used. Finally, the color development was stopped, and the intensity of the color was measured using a microplate reader at 450 nm.

### Culture and Treatment of 3T3-L1 Cells

3T3-L1 cell line was purchased from ATCC (Manassas, VA, USA). Cells were cultured, tested for mycoplasma contamination, and differentiated into adipocytes according to the protocol described by Guzman-Ruiz et al. (19). Differentiated cells were used only when at least 90% showed an adipocyte phenotype by accumulation of lipid droplets by day 8. On day 8 of differentiation, 3T3-L1 adipocytes were treated for 24 h with medium containing 10% inactivated serum (incubated at 56°C for 30 min) from six healthy donors and six RA patients with moderate–high disease activity, alone or combined with leflunomide (LFN), methotrexate (MTX), or hydroxychloroquine (HCQ) (50 μM). Subsequently, cells were collected for mRNA analyses.

### RNA Extraction and Gene Expression

RNA from gonadal adipose tissue of mice was extracted using TRI Reagent (Sigma) according to the manufacturer’s recommendations and reverse transcribed to cDNA. Real-Time PCR using SYBR green or Taqman was performed according to the manufacturer’s instructions (ABI). Expression of genes of interest was corrected by the geometrical average of 18s, β2m, β-actin, and 36b4 using the Bestkeeper tool (20). The expression of genes involved in inflammation (Interleukin 1β, IL-1β; TNF-α), macrophage presence and polarization (EGF-like module containing, mucin-like, hormone receptor-like sequence, F4/80; Integrin subunit alpha X, CD11c and macrophage mannose receptor, CD206), adipogenesis (Peroxisome Proliferator Activated Receptor gamma, PPARγ, and Fatty acid Synthase, FAS), lipolysis (Hormone-Sensitive Lipase, HSL, and Adipose triglyceride lipase, ATGL), and insulin and glucose signaling (Glucose transporter type 4, GLUT-4; Insulin receptor substrate 1, IRS-1 and Insulin receptor substrate 2, IRS-2) were analyzed.

### Statistical Analysis

Student t-test (unpaired), ANOVA, and Duncan test were used for the statistical analysis. Statistically significance was set at p<0.05 and p<0.01. Spearman correlation was calculated to estimate the linear correlations between variables p<0.01.

## Results

### Obesity Does Not Increase the Activity of the Disease in Established RA

Among our cohorts of RA patients, the age, duration of the disease, and autoantibodies’ presence were not significantly different between the obese and lean patients. Of note, RA obese patients had significantly less number of tender and swollen joints compared to RA lean patients. VAS was also significantly decreased in obese RA patients. No changes were noticed in ESR between the two RA groups, which was translated into a reduction in the disease activity index (DAS28-ESR) in obese RA patients. However, levels of C reactive protein (CRP) were significantly increased in the obese RA patients compared to non-obese-RA patients. This elevation was mainly due to the fat burden, as observed in the CRP levels of obese subjects with no RA (**Table 1**). The elevation of CRP due to the increased weight in obese RA patients might be responsible for the values of DAS28 (CRP). Regarding the glucose and lipid profile, triglyceride levels were elevated in both groups of RA patients comparing to healthy donors. In addition, obese RA patients had increased levels of triglycerides *vs* lean RA patients, similar to what was observed in obese non-RA subjects. Fasting glucose levels were elevated in obese individuals, RA and non-RA (**Table 1**). In sum, lipid and glucose components were not different between obese subjects, including those with RA and without the disease. These results suggest that these alterations were only due to the increased body mass index (BMI).

### Obesity Accelerates the Onset of Arthritis, but Does Not Aggravate the Disease Course in Arthritic Mice

**Figure 1:**
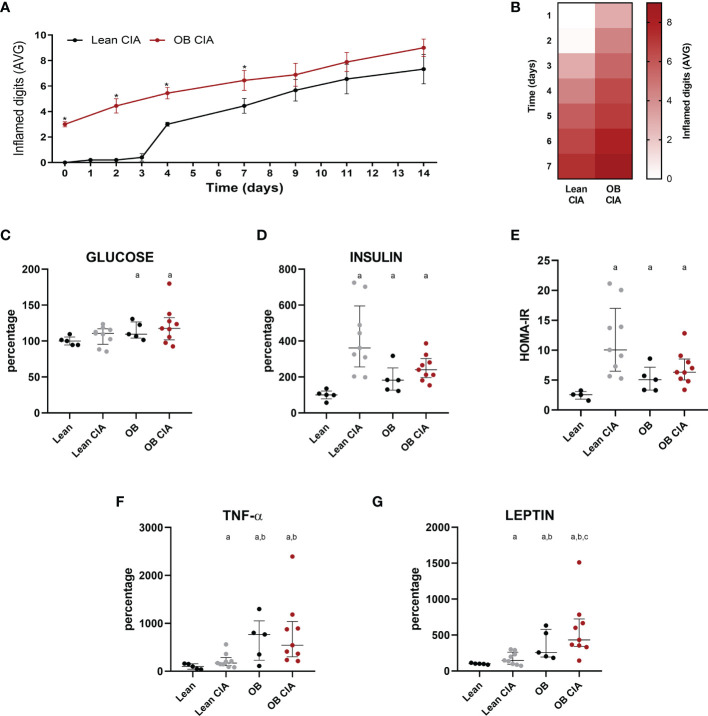
Obesity accelerates the arthritis onset: inflammatory and metabolic changes in mice. **(A)** Mean number of inflamed digits during disease progression in lean and OB CIA mice. Significance was determined by Student t-test (unpaired), **(B)** Heatmap showing the mean number of inflamed digits during arthritis progression in lean and OB CIA mice. **(C)** Plasma glucose levels in lean, lean CIA, obese, and obese CIA mice, **(D)** Plasma insulin levels in lean, lean CIA, obese, and obese CIA mice, **(E)** HOMA-IR of lean, lean CIA, obese, and obese CIA mice, **(F)** Plasma TNF-α levels in lean, lean CIA, obese, and obese CIA mice, and **(G)** Plasma leptin levels of lean, lean CIA, obese, and OB CIA mice. CIA, collagen induced arthritis; OB, obese; HOMA-IR, homeostatic model assessment–insulin resistance; TNF-α, tumor necrosis factor alpha. Significance was determined by one-way ANOVA followed by Ducan *post hoc* test **(C–G)**. *Significant differences *vs.* lean CIA (p < 0.05). ^a^Significant differences *vs.* lean (p < 0.05). ^b^Significant differences *vs.* lean CIA (p < 0.05). ^c^Significant differences *vs.* OB (p < 0.05).

CIA obese mice had already inflamed joints by the time of collagen induction due to the joint overload. Thus, generation of arthritis was more pronounced in obese mice from the onset of the disease until day 9. However, at day 9 after induction, there were no differences in the number of inflamed joints between lean and obese CIA mice (**Figures 1A, B**).

### Insulin Resistance in Obese and Lean Arthritic Mice

High levels of glucose were detected only under obesity conditions [obese (OB) and OB CIA mice] (**Figure 1C**). However, plasma insulin levels were significantly increased in lean CIA, OB, and OB CIA mice, which was translated into an increase in HOMA-IR in these three groups of mice, suggesting that IR in arthritis is due to the high levels of insulin (**Figures 1D, E**). These levels of HOMA-IR were accompanied by high levels of circulating TNF-α, which were more increased under obesity conditions (**Figure 1F**). Circulating levels of leptin were significantly increased in CIA and obese mice. Among them, the simultaneous presence of obesity and arthritis elevated the leptin levels compared to the groups having obesity or arthritis alone (**Figure 1G**).

### Molecular Impact of Arthritis in Inflammation and Lipid/Glucose Metabolisms of Adipose Tissue in Lean and Obese Mice

**Figure 2:**
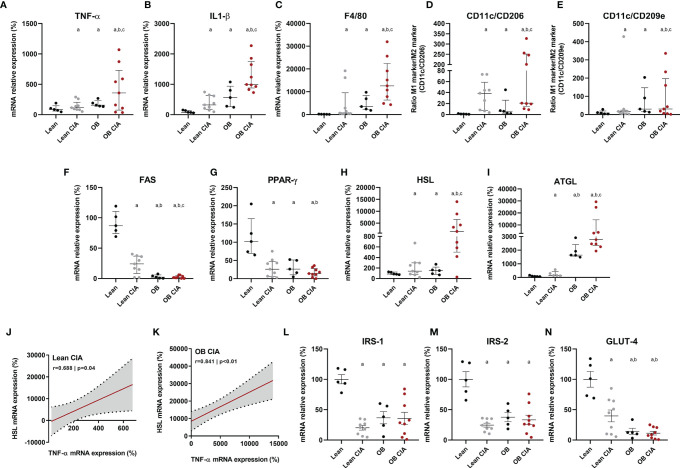
Inflammatory and metabolic profile in adipose tissue of OB CIA mice. Inflammation, presence and polarization of macrophages, and glucose and lipid metabolisms. **(A)** mRNA relative expression of TNF-α in white adipose tissue of control lean, Lean CIA, OB, and OB CIA, **(B)** mRNA relative expression of IL-1β white adipose tissue of control lean, Lean CIA, OB, and OB CIA, **(C)** mRNA relative expression of F4/80 in white adipose tissue of control lean, Lean CIA, OB, and OB CIA, **(D)** ratio of CD11c/CD206 mRNA relative expression in white adipose tissue of control lean, Lean CIA, OB, and OB CIA, **(E)** ratio of CD11c/CD209e mRNA relative expression in white adipose tissue of control lean, Lean CIA, OB, and OB CIA, **(F)** mRNA relative expression of FAS in white adipose tissue of control lean, Lean CIA, OB, and OB CIA, **(G)** mRNA relative expression of PPARγ in white adipose tissue of control lean, Lean CIA, OB, and OB CIA, **(H)** mRNA relative expression of HSL in white adipose tissue of control lean, Lean CIA, OB, and OB CIA, **(I)** mRNA relative expression of ATGL in white adipose tissue of control lean, Lean CIA, OB, and OB CIA, **(J)** correlation between the mRNA expression of HSL and TNF-α in white adipose tissue of lean CIA, **(K)** correlation between the mRNA expression of HSL and TNF-α in white adipose tissue of OB CIA, **(L)** mRNA relative expression of IRS-1 in white adipose tissue of control lean, Lean CIA, OB, and OB CIA, **(M)** mRNA relative expression of IRS-2 in white adipose tissue of control lean, Lean CIA, OB, and OB CIA, and **(N)** mRNA relative expression of GLUT-4 in white adipose tissue of control lean, Lean CIA, OB, and OB CIA. CIA, collagen induced arthritis; OB, obese; TNF-α, tumor necrosis factor alpha; IL-1β, interleukin 1 beta; F4/80, cell surface glycoprotein F4/80; CD, cluster differentiation; FAS, fatty acid synthase; PPARγ, Peroxisome Proliferator Activated Receptor Gamma; HSL, hormone sensitive lipase; ATGL, adipose triglyceride lipase; IRS-1, insulin receptor substrate 1; GLUT-4, glucose transporter type 4. Significance was determined by one-way ANOVA followed by Ducan *post hoc* test **(A–I, L–N)**. ^a^Significant differences *vs.* lean (p < 0.05). ^b^Significant differences *vs.* lean CIA (p < 0.05). ^c^Significant differences *vs.* OB (p < 0.05).

**Figure 3:**
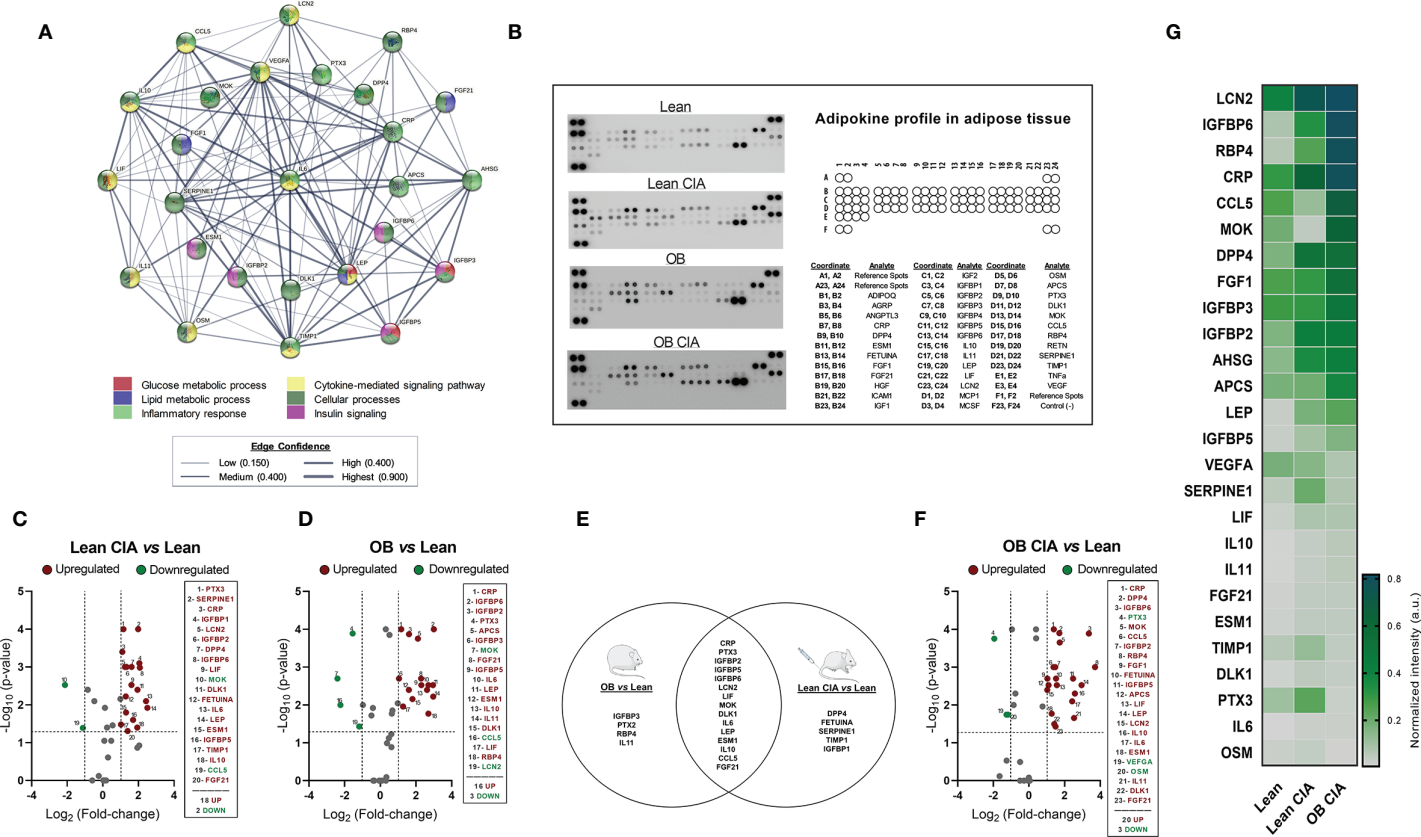
Adipokine profiling in adipose tissue of OB CIA mice. **(A)** Bioinformatic identification of biological functions of profiling proteins in adipose tissue (STRING platform); **(B)** Representative images of mouse adipokine arrays and the coordinate reference of proteins; **(C)** Volcano plot of proteins altered in Lean CIA mice compared to Lean mice; **(D)** Volcano plot of proteins altered in OB mice compared to lean mice; **(E)** Venn diagram of common and non-common proteins altered in Lean CIA and OB mice; **(F)** Volcano plot of proteins altered in OB CIA mice compared to lean mice; **(G)** Heatmap of mean pixel density of adipokine profile. Volcano plots compare the fold change and p-value of the expression of proteins between groups. Vertical dashed mark the fold change (>1) and horizontal lines mark the p-value cut-off of 0.05. Significance was determined by Student t-test (unpaired). ADIPOQ, adiponectin; AGRP, agouti-related neuropeptide; ANGPTL3, angiopoietin like 3; CRP, c-reactive protein; DPP4, dipeptidyl peptidase 4; ESM1, endothelial cell specific molecule 1; FGF, fibroblast growth factor; HGF, hepatocyte growth factor; ICAM1, intercellular adhesion molecule 1; IGF, insulin like growth factor; IGFBP, insulin-like growth factor binding protein; IL, interleukin; LEP, leptin; LIF, leukemia inhibitory factor; LCN2, lipocalin-2; MCP1, monocyte chemoattractant protein-1; MCSF, macrophage colony stimulating factor-1; OSM, oncostatin M; APCS, amyloid P component serum; PTX3, pentraxin 3; DLK1, delta like non-canonical notch ligand 1; MOK, MAPK overlapping kinase; CCL5, C-C motif chemokine ligand 5; RBP4, retinol binding protein 4; RETN, resistin; SERPINE1, serpin family E receptor member 1; TIMP1, tissue inhibitor of metalloproteinases 1; TNF-α, tumor necrosis factor alpha; VEGF, vascular endothelial growth factor; CIA, collagen induced arthritis; OB, obese; MTX, methotrexate; LFN, leflunomide; HCQ, hydroxychloroquine.

The expression of several inflammatory mediators was measured in adipose tissue from obese and CIA mice. Thus, mRNA levels of TNF-α and IL-1β were significantly increased in lean CIA mice and obese mice compared to lean control group. In addition, those mice having both, obesity and arthritis diseases, displayed elevated levels of these cytokines compared to CIA lean and control OB mice (**Figures 2A, B**). The expression of genes related to macrophage infiltration (F4/80) and the ratio of mRNA expression of genes involved in macrophage M1 polarization state (CD11c/CD206 and CD11c/CD209e) were significantly elevated in arthritic mice compared to control lean, suggesting that arthritis induces the infiltration and presence of M1 macrophages in adipose tissue (**Figures 2C–E**). Thus, similarly to what was observed in the expression of inflammatory molecules, the expression of genes related to macrophage infiltration and polarization was significantly increased in those mice having the two conditions, obesity and arthritis, compared to those displaying only one disease (obesity or arthritis) (**Figures 2C–E**). Regarding lipid metabolism, the induction of both disorders, arthritis and obesity, alone or combined, promoted a decrease in the mRNA expression of genes involved in lipid accumulation (FAS and PPARγ) and an increase in the expression of genes related to lipolysis, such as HSL and ATGL (**Figures 2F–I**). The changes promoted in the expression of those genes were greater in the mice having both conditions, obesity and arthritis. In addition, those effects seemed to be induced by the inflammatory burden as observed in the elevated levels of inflammatory mediators and the correlation between mRNA expression of TNF-α and HSL in lean and OB CIA mice (**Figures 2J, K**). Likewise, levels of genes involved in glucose metabolism such as IRS-1, IRS-2, and GLUT-4 were reduced upon obesity or arthritis induction, either alone or combined, which may facilitate an insulin resistance state (**Figures 2L–N**). After analyzing the proteome profile of adipose tissue, the induction of arthritis promoted an alteration in the expression of proteins involved in lipid and glucose metabolisms and inflammation (**Figure 3A**). Thus, 20 out of the 38 proteins analyzed were modified in adipose tissue from Lean CIA mice compared to controls (**Figures 3B, C**). Interestingly, 15 of these proteins were commonly altered in the adipose tissue, after comparing obese *versus* lean mice (**Figures 3B, D, E**), suggesting that arthritis may induce adipose tissue dysfunction, similar to what it could be expected upon obesity conditions, regardless the lipid accumulation. In addition, the coexistence of CIA and obesity induced a major change in the adipose tissue, altering the expression of 23 proteins compared to lean mice (**Figures 3F, G**).

### Effects of Conventional DMARDs in Inflammation, Body Weight, and Insulin Resistance in Obese Arthritic Mice

**Figure 4:**
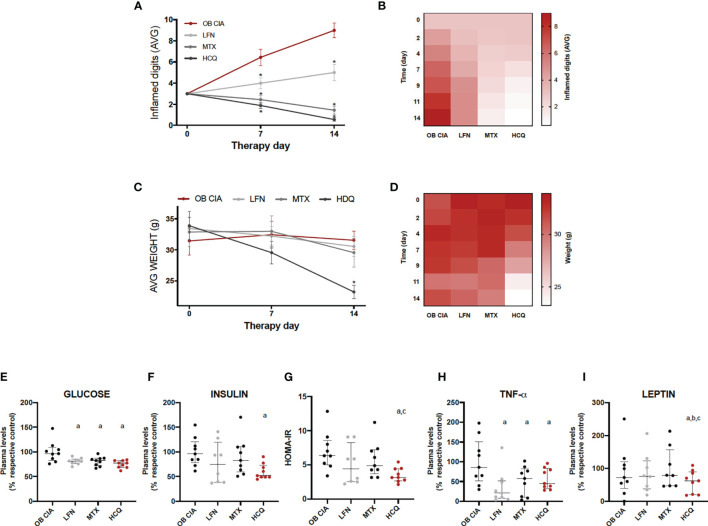
Effect of cDMARDs in systemic inflammation, body weight, and insulin resistance of OB CIA mice. **(A)** Mean number of inflamed digits in OB CIA mice treated with LFN, MTX, or HCQ. Significance was determined by Student t-test (unpaired). *Significant differences *vs.* OB CIA (p < 0.05). **(B)** Heatmap showing the mean number of inflamed digits of OB CIA mice treated with LFN, MTX, and HCQ. **(C)** Body weight of OB CIA mice treated with LFN, MTX, and HCQ. **(D)** Heatmap showing the body weight of OB CIA mice treated with LFN, MTX, and HCQ. Significance was determined by Student t-test (unpaired). *Significant differences *vs.* OB CIA (p < 0.05). **(E)** Plasma glucose levels in OB CIA mice after 14 days of treatment with LFN, MTX, and HCQ. **(F)** Plasma insulin levels in OB CIA mice after 14 days of treatment with LFN, MTX, and HCQ. **(G)** HOMA-IR of OB CIA mice after 14 days of treatment with LFN, MTX, and HCQ. **(H)** Plasma TNF-α levels of OB CIA mice after 14 days of treatment with LFN, MTX, and HCQ. **(I)** Plasma leptin levels of OB CIA mice after 14 days of treatment with LFN, MTX, and HCQ. OB, obese; CIA, collagen-induced arthritis; LFN, leflunomide; MTX, methotrexate; HCQ, hydroxychloroquine; HOMA-IR, homeostatic model assessment–insulin resistance; TNF-α, tumor necrosis factor alpha. ^a^Significant differences *vs.* OB CIA (p < 0.05). ^b^Significant differences *vs.* LFN (p < 0.05). Significance was determined by one-way ANOVA followed by Ducan *post hoc* test **(E–I)**. ^c^Significant differences *vs.* MTX (p < 0.05).

The effects of the three cDMARDs studied (LFN, MTX, and HCQ) preventing the inflammation in the digits of obese arthritic mice were significantly noticeable from day 2 of treatment. In addition, the most effective treatments were MTX and HCQ compared to LFN (**Figures 4A, B**). However, HCQ was the only treatment that had the capacity of reducing the body weight, being significant from day 9 of treatment (**Figures 4C, D**). Although the three treatments decreased plasma glucose levels in our model of obese arthritic mice, only HCQ had a beneficial effect lowering the levels of insulin and, thus, reducing insulin resistance (**Figures 4E–G**). In addition, plasma levels of both TNF-α and leptin were reduced after 14 days of treatment with either cDMARD, with no differences among them (**Figure 4H**). However, HCQ was the only treatment able to reduce the plasma levels of leptin (**Figure 4I**). These results suggest that three cDMARDs are able to control the systemic inflammation, but only HCQ displays a metabolic action, improving HOMA-IR and reducing weight.

### Molecular Effects of cDMARDs in Adipose Tissue of Arthritic Obese Mice

**Figure 5:**
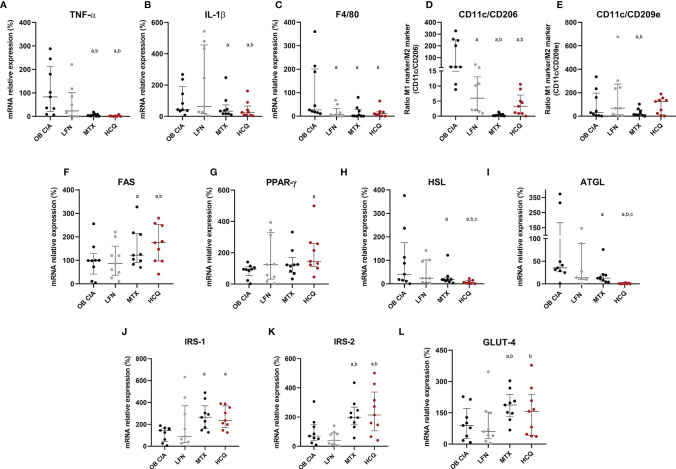
Molecular effects of cDMARDs in white adipose tissue of OB CIA mice. **(A)** mRNA relative expression of TNF-α in white adipose tissue of OB CIA mice after 14 days of treatment with LFN, MTX, and HCQ. **(B)** mRNA relative expression of IL-1β in white adipose tissue of OB CIA mice after 14 days of treatment with LFN, MTX, and HCQ. **(C)** mRNA relative expression of F4/80 in white adipose tissue of OB CIA mice after 14 days of treatment with LFN, MTX, and HCQ. **(D)** ratio of CD11c/CD206 mRNA relative expression in white adipose tissue of OB CIA mice after 14 days of treatment with LFN, MTX, and HCQ. **(E)** ratio of CD11c/CD209e mRNA relative expression in white adipose tissue of OB CIA mice after 14 days of treatment with LFN, MTX, and HCQ. **(F)** mRNA relative expression of FAS in white adipose tissue of OB CIA mice after 14 days of treatment with LFN, MTX, and HCQ. **(G)** mRNA relative expression of PPARg in white adipose tissue of OB CIA mice after 14 days of treatment with LFN, MTX, and HCQ. **(H)** mRNA relative expression of HSL in white adipose tissue of OB CIA mice after 14 days of treatment with LFN, MTX, and HCQ. **(I)** mRNA relative expression of ATGL in white adipose tissue of OB CIA mice after 14 days of treatment with LFN, MTX, and HCQ. **(J)** mRNA relative expression of IRS-1 in white adipose tissue of OB CIA mice after 14 days of treatment with LFN, MTX, and HCQ. **(K)** mRNA relative expression of IRS-2 in white adipose tissue of OB CIA mice after 14 days of treatment with LFN, MTX, and HCQ. **(L)** mRNA relative expression of GLUT-4 in white adipose tissue of OB CIA mice after 14 days of treatment with LFN, MTX, and HCQ. OB, obese; CIA, collagen-induced arthritis; LFN, leflunomide; MTX, methotrexate; HCQ, hydroxychloroquine; TNF-α, tumor necrosis factor alpha; IL-1β, interleukin 1 beta; F4/80, cell surface glycoprotein F4/80; CD, cluster differentiation; FAS, fatty acid synthase; PPARγ, peroxisome proliferator activated receptor gamma; HSL, hormone sensitive lipase; ATGL, adipose triglyceride lipase; IRS-1, insulin receptor substrate 1; IRS-2, insulin receptor substrate 2; GLUT-4, glucose transporter type 4. Significance was determined by one-way ANOVA followed by Ducan *post hoc* test. ^a^Significant differences *vs.* OB CIA (p < 0.05). ^b^Significant differences *vs.* LFN (p < 0.05). ^c^Significant differences *vs.* MTX (p < 0.05).

**Figure 6:**
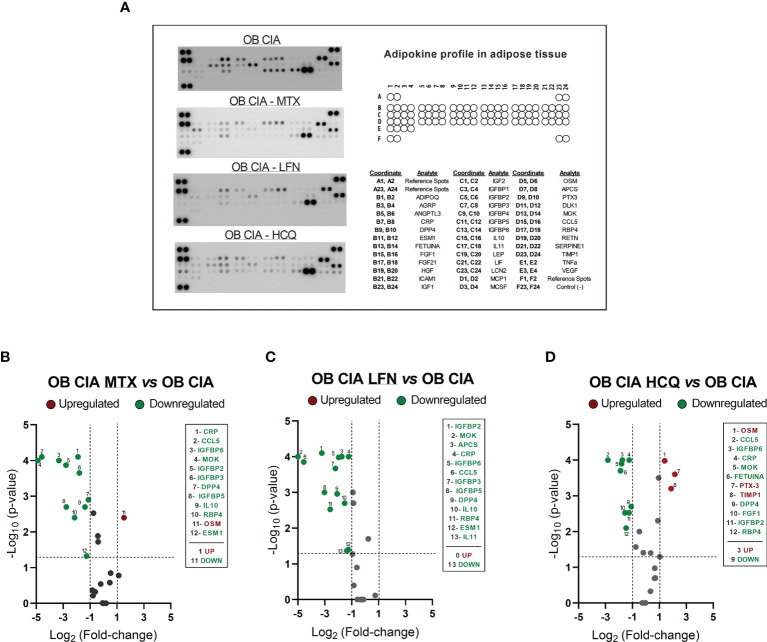
Adipokine profiling in adipose tissue of OB CIA mice treated with conventional DMARDs. **(A)** Representative images of mouse adipokine array and the coordinate reference of proteins; **(B)** Volcano plot of modified proteins in OB CIA mice treated with MTX; **(C)** Volcano plot of modified proteins in OB CIA mice treated with LFN; **(D)** Volcano plot of modified proteins in OB CIA mice treated with HCQ. Volcano plots compare the fold change and p-value of the expression of proteins between groups. Vertical dashes mark the fold change (>1), and horizontal lines mark the p-value cutoff of 0.05. Significance was determined by Student t-test (unpaired). ADIPOQ, adiponectin; AGRP, agouti-related neuropeptide; ANGPTL3, angiopoietin like 3; CRP, C-reactive protein; DPP4, dipeptidyl peptidase 4; ESM1, endothelial cell specific molecule 1; FGF, fibroblast growth factor; HGF, hepatocyte growth factor; ICAM1, intercellular adhesion molecule 1; IGF, insulin like growth factor; IGFBP, insulin-like growth factor binding protein; IL, interleukin; LEP, leptin; LIF, leukemia inhibitory factor; LCN2, lipocalin-2; MCP1, monocyte chemoattractant protein-1; MCSF, macrophage colony stimulating factor-1; OSM, oncostatin M; APCS, amyloid P component serum; PTX3, pentraxin 3; DLK1, delta like non-canonical notch ligand 1; MOK, MAPK overlapping kinase; CCL5, C-C motif chemokine ligand 5; RBP4, retinol binding protein 4; RETN, resistin; SERPINE1, serpin family E receptor member 1; TIMP1, tissue inhibitor of metalloproteinases 1; TNF-α, tumor necrosis factor alpha; VEGF, vascular endothelial growth factor; CIA, collagen-induced arthritis; OB, obese; MTX, methotrexate; LFN, leflunomide; HCQ, hydroxychloroquine.

The effects of cDMARDs in the expression of inflammatory mediators in adipose tissue were analyzed. Thus, white adipose tissue from obese arthritic mice showed reduced levels of TNF-α and IL-1β after treatment with MTX or HCQ (**Figures 5A, B**). Regarding markers of macrophage infiltration, treatment with the three cDMARDs significantly lowered the mRNA expression of F4/80 in adipose tissue, a specific marker of macrophages, suggesting a reduction in the macrophage infiltration (**Figure 5C**). Likewise, the expression of markers of M1 macrophage state was also reduced, MTX being the most effective treatment causing the greater change in the expression (**Figures 5D, E**). Regarding the expression of genes involved in lipid and glucose metabolisms, MTX and HCQ were the treatments that regulated the mRNA expression of FAS, PPARγ, HSL, ATGL, IRS-1, IRS-2, and GLUT4 (**Figures 5F–L**). We also observed a significant effect of cDMARDs in the expression of proteins in the adipose tissue of obese CIA mice. In fact, the three drugs had a noticeable impact in the adipokine profile (**Figures 6A–D**).

### Specific Effects of cDMARDs in Adipocyte-Activated RA Serum

**Figure 7:**
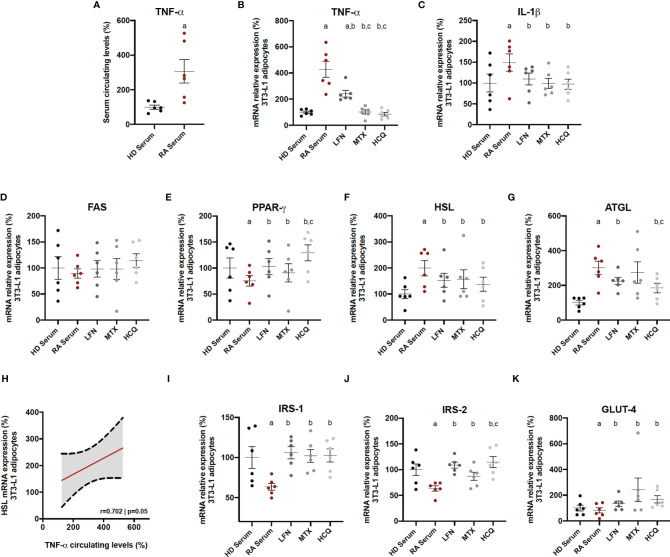
*In vitro* effects of cDMARDs in adipocyte-activated RA serum. **(A)** Levels of TNF-α in serum from six healthy donors and six RA patients. **(B)** mRNA relative expression of TNF-α in 3T3-L1-derived adipocytes after 24 h of treatment with LFN, MTX, and HCQ. **(C)** mRNA relative expression of IL-1β in 3T3-L1 derived adipocytes after 24 h of treatment with LFN, MTX, and HCQ. **(D)** mRNA relative expression of FAS in 3T3-L1-derived adipocytes after 24 h of treatment with LFN, MTX, and HCQ. **(E)** mRNA relative expression of PPARγ in 3T3-L1 derived adipocytes after 24 h of treatment with LFN, MTX, and HCQ. **(F)** mRNA relative expression of HSL in 3T3-L1-derived adipocytes after 24 h of treatment with LFN, MTX, and HCQ. **(G)** mRNA relative expression of ATGL in 3T3-L1-derived adipocytes after 24 h of treatment with LFN, MTX, and HCQ. **(H)** Correlation between the mRNA expression of HSL in 3T3-L1 adipocytes and the levels of TNF-α in the RA serum. **(I)** mRNA relative expression of IRS-1 in 3T3-L1-derived adipocytes after 24 h of treatment with LFN, MTX, and HCQ. **(J)** mRNA relative expression of IRS-2 in 3T3-L1-derived adipocytes after 24 h of treatment with LFN, MTX, and HCQ. **(K)** mRNA relative expression of GLUT-4 in 3T3-L1-derived adipocytes after 24 h of treatment with LFN, MTX, and HCQ. HD, healthy donor; RA, rheumatoid arthritis; TNF-α, tumor necrosis factor alpha; IL-1β, interleukin 1 beta; FAS, fatty acid synthase; PPARγ, peroxisome proliferator activated receptor gamma; HSL, hormone sensitive lipase; ATGL, adipose triglyceride lipase; IRS-1, insulin receptor substrate 1; IRS-2, insulin receptor substrate 2; GLUT-4, glucose transporter type 4. Significance was determined by one-way ANOVA followed by Ducan *post hoc* test **(A–G, I–K)**. ^a^Significant differences *vs.* treatment with HD serum (p < 0.05). ^b^Significant differences *vs.* treatment with LFN (p < 0.05). ^c^Significant differences *vs.* treatment with MTX (p < 0.05).

The specific *in vitro* effects of the three cDMARDs on adipocytes in a context of RA were evaluated. For that purpose, adipocytes were firstly induced with serum (10%) from RA patients, which contained a high amount of TNF-α (**Figure 7A**). Thus, RA serum treatment induced the mRNA expression of inflammatory mediators, such as TNF-α and IL-1β, on 3T3-L1 adipocytes (**Figures 7B, C**). The addition of LFN, MTX, or HCQ significantly reduced the levels of these cytokines, although MTX and HCQ showed superiority in the reduction of the mRNA expression of TNF-α compared to the treatment with LFN (**Figures 7B, C**). Regarding the expression of genes related to lipid metabolism, the three cDMARDs restored the levels of PPARγ, HSL, and ATGL altered by the treatment with RA serum (**Figures 7D–G**). In addition, the high levels of TNF-α in the serum from RA patients correlated with the increase in the mRNA expression of HSL in 3T3-L1 adipocytes, suggesting the direct effect of TNF-α in the regulation of this hormone (**Figure 5H**). Finally, LFN, MTX, and HCQ also increased the mRNA expression of IRS-1, IRS-2, and GLUT-4 on adipocytes reduced by the treatment with RA serum (**Figures 7I–K**). Some of these genes involved in lipid and glucose metabolisms (PPARγ, ATGL, and IRS2) were even more regulated by HCQ (**Figures 7E, G, J**).

## Discussion

This study shows for the first time the molecular changes that arthritis could promote in adipose tissue, and adds clarification about the association between RA and obesity, using human cohorts of obese and lean RA patients and a model of obese arthritic mouse. In addition, the effects of conventional DMARDs in inflammation and molecular alterations in adipose tissue and specifically in the adipocyte are also described.

Interestingly, in our cohort of RA patients with low-moderate disease activity, the presence of obesity was not related to higher activity of the disease, and DAS28-CRP was slightly higher in obese RA patients compared to non-obese but with no statistical difference. In fact, the number of swollen and tender joints was significantly decreased in the obese RA patients compared to the lean patients. Of note, we could observe that the increase of CRP levels in obese RA patients was mainly due to the fat burden more than the RA itself since those levels in obese subjects were increased as well, not finding differences between obese RA patients and obese non-RA. On the other hand, ESR levels were unchanged between lean and obese RA patients, which led to a significant reduction in the DAS28-ESR in RA obese patients. These data suggest that the disease activity in obese RA patients should be carefully measured due to the increase of the CRP by the augmentation of BMI.

Clinical disease activity is frequently observed to be high in obese patients with RA (9, 15); however, some authors did not associate obesity with disease activity (21, 22), which is in line with our data. In addition, there is data showing a protective action of obesity for radiographic joint damage. The mechanisms proposed comprise several phenomena including stimulation of bone synthesis by the elevated mechanical loading, the higher levels of estrogens in obese RA patients and adiponectin (23). Regarding adiponectin, there is controversial evidence about its role in RA, due to the fact of dual effects, having both anti-inflammatory and pro-inflammatory effects (24). Adiponectin, which is reduced in obese people, is an adipokine that in the context of RA plays an inflammatory role correlating with disease activity in lean RA patients (25) and can potentiate joint damage (26). However, the reduced levels in obese RA due to the fat burden might prevent its pro-inflammatory effects in the joint.

Sharma et al. recently reported similar observations; they claimed that RA obese patients may have elevated inflammatory markers (CRP and ESR), mainly because of obesity (27). Additionally, these authors, alongside George et al., found that CRP and ESR levels positively correlated with obesity in females, but not in males (28). This fact might be due to the possible inhibitory effect of testosterone in CRP levels (29).

Our results are in line with these studies, supporting the concept that obesity may falsely increase the levels of CRP in RA patients with low disease activity, giving elevated rates of disease activity in those patients. Clinicians should be cautious measuring the activity of the RA in obesity.

Regarding insulin resistance in RA, HOMA-IR values are elevated in RA patients regardless of obesity as we have previously described, where inflammatory mediators elevated in RA might be the main actors in the development of IR in the context of RA (30). In the present study, we show that the presence of both conditions, obesity and RA, did not increase the HOMA-IR values and the levels of triglycerides elevated due to the obesity, suggesting that RA does not aggravate IR or the alteration of the lipid profile in an obesity scenario.

The data observed in the human cohorts was confirmed in the CIA mice. Thus, after 15 days of the arthritis induction, obesity did not significantly affect the number of inflamed digits compared to the lean mice. Likewise, plasma levels of TNF-α, serum insulin and HOMA-IR were similar in both groups of CIA mice, obese and lean. Up to date, there are scarce studies performed in this mouse model of obese arthritis. In one of them, it has been suggested that obesity plays an important role in the progression of RA, due to the joint overload, promoting the production of inflammatory molecules, such as IL-17 in the synovium, damaging the tissue (31). On the other hand, it has been demonstrated that obesity aggravates the inflammation in the digits of CIA mice, affecting critically the early stages of the disease. However, after 30 days of CIA disease progression, joint swelling, infiltrated leukocytes, and macrophage differentiation were similar between obese and lean CIA mice (32).

This is the first study that shows the transcriptomic changes of the white adipose tissue induced by arthritis in a context of obesity. Thus, the alteration in the expression of genes related to inflammation, M1 macrophage presence, and polarization markers and lipid metabolism was greater in those mice having both conditions, obesity and arthritis. In addition, the elevated expression of TNF-α was directly correlated with the increase of HSL, supporting the fact that TNF-α stimulates lipolysis (33). Increased presence of macrophages in the subcutaneous adipose tissue of RA patients and its relationship with the anti-CCPs positivity have recently been described (34). It is well known that obesity is associated with macrophage accumulation in the adipose tissue in a pro-inflammatory M1 state, characterized by the expression of inflammatory markers contributing to the IR (35). Our data in an obese arthritic mouse model showed that markers of macrophage presence and M1 polarization state were elevated in the mice displaying both conditions, obesity and arthritis.

All the changes in transcriptomics at tissue level were accompanied by an increase in the circulating levels of leptin in OB CIA mice reflecting the abnormal function of the adipose tissue. Leptin is an inflammatory adipokine with a significant role in the immunopathogenesis of RA (36). Its functions comprise the stimulation of the production of inflammatory cytokines, such as TNF-α and the regulation of the glucose metabolism among others, contributing to the development of IR.

These results suggest that arthritis affects directly the inflammatory burden and metabolic alterations associated with obesity in adipose tissue, aggravating the dysfunction of this tissue. Our study also shows for the first time the effect of cDMARDs in the adipose tissue alterations associated with RA. First of all, our work performed in obese CIA mice confirms previous studies that suggest that MTX and HCQ are more effective than LFN in reducing inflammation, as it is indicated by the EULAR (European League Against Rheumatism) recommendations (37). HCQ was the treatment that reduced the body weight of the obese CIA mice, accompanied by an improvement in the insulin sensitivity. These results support recent studies that described that HCQ reduced the IR state and improved the glucose homeostasis in CIA rats (38). A number of studies conducted in longitudinal and cross-sectional cohorts claimed that the use of HCQ reduces the incidence of diabetes in RA patients (39, 40). Beneficial effects of HCQ improving insulin sensitivity have been extended to non-rheumatic obese patients with IR (41). Our data support the concept that HCQ could have positive effects in the IR state of obese RA patients. Besides the accumulating data about the effect of this antimalarial on glucose and insulin metabolism, specific impact on adipose tissue in the RA scenario has not been described yet.

As it be could expected, the treatment of obese CIA mice with either of the three cDMARDs reduced the expression of markers related to inflammation, macrophage presence, and M1 polarization state in the adipose tissue. In this sense, a recent study showed that subcutaneous adipose tissue from RA patients taking MTX or LFN had lower levels of adipose tissue macrophages and crown-like structures (34).

Interestingly, treatment of CIA mice with HCQ or MTX had also a positive effect in the adipose tissue, increasing the expression of genes involved in the insulin signaling, lipogenesis, and lipid accumulation and reducing those related to lipolysis, alterations induced by the induction of arthritis as we previously showed (30). These results were confirmed through *in vitro* experiments with adipocytes treated with serum from RA combined with the cDMARDs. There are few evidences about the effect of MTX in the metabolic comorbidities. A recent study described that RA patients taking MTX were less prone to suffer metabolic syndrome, since they displayed an improvement in the insulin sensitivity through the increase of the insulin activity and the transport and metabolism of glucose (42).

Little has been reported about the effect of LFN in lipid and glucose metabolisms. Chen et al. recently showed that LFN was able to control hyperglycemia and IR in two obese mice models, although these effects were not noticed in lean mice (43). In our obese CIA mice, LFN reduced blood glucose levels, but no effects were observed in the HOMA-IR values. One study reported that LFN decreased leptin levels in mice with dyslipidemia and lipodystrophy (44). In our hands, LFN was able to reduce plasma TNF-α levels, but levels of leptin were unchanged. In addition, the subcutaneous adipose tissue of obese CIA mice had decreased inflammation, but no differences in the expression of genes involved in lipid accumulation, lipolysis, and insulin signaling were observed after treatment with LFN. However, LFN was capable of modulating those genes in 3T3-L1 adipocytes with RA serum, which was in agreement with Chen et al., who showed that LFN *in vitro* enhanced insulin receptor signaling and increased glucose uptake in 3T3-L1 adipocytes (43). Additionally, a clinical study revealed that blood glucose levels of RA patients treated with LFN at dose of 83 mg/L were lower comparing with other regimens of LFN (45). Data about the beneficial effects of LFN on glucose metabolism and insulin sensitivity are inconsistent; thus, further studies need to be performed to clearly define the mechanism of action of this drug in both lipid and glucose metabolisms.

MTX is the most effective cDMARD controlling inflammation. We here show that it also has positive effects regulating genes involved in lipid and glucose homeostasis in the adipose tissue, although these could be limited. On the other hand, HCQ has a limited efficacy for disease activity and progression, being not usually used as monotherapy for the treatment of RA. However, HCQ may benefit the metabolic profile in patients with RA, acting at systemic and adipose tissue levels. Our study performed in obese CIA mice treated with cDMARDs suggests the usefulness of the combined treatment of HCQ and MTX in the metabolic alterations induced by obesity at both systemic and adipose tissue levels in RA.

## Data Availability Statement

The original contributions presented in the study are included in the article/supplementary material. Further inquiries can be directed to the corresponding authors.

## Ethics Statement

The studies involving human participants were reviewed and approved by the Reina Sofia Hospital Ethics Committee. The patients/participants provided their written informed consent to participate in this study. The animal study was reviewed and approved by the biosafety and bioethics committee of University of Cordoba.

## Author Contributions

Conceptualization, supervision, writing—original draft preparation, writing—review and editing, project administration, funding acquisition: NB, CL-P, AE-C, RG-R, MMM, FT, and EC-E. Methodology, software, validation, formal analysis, data curation: IAR, MR-P, CP-S, CR-R, AP-T, ML-T, MA-A, and JA. Data curation and followed up with patients: MM, RO-C, JC-G, and PF.

## Funding

This research was funded by grants from the Instituto de Salud Carlos III (PI17/01316 and PI18/00837), co-financed by the European Regional Development Fund (ERDF), a way to make Europe, Spain, MINECO (RyC-2017-23437), and the Spanish Inflammatory and Rheumatic diseases Network (RIER, RD16/0012/0015). CL-P was supported by a contract from the Junta de Andalucia (Nicolas Monardes programme).

## Conflict of Interest

The authors declare that the research was conducted in the absence of any commercial or financial relationships that could be construed as a potential conflict of interest.

## Publisher’s Note

All claims expressed in this article are solely those of the authors and do not necessarily represent those of their affiliated organizations, or those of the publisher, the editors and the reviewers. Any product that may be evaluated in this article, or claim that may be made by its manufacturer, is not guaranteed or endorsed by the publisher.

## References

[1] Del Rincon ID Williams K Stern MP Freeman GL Escalante A. High Incidence of Cardiovascular Events in a Rheumatoid Arthritis Cohort Not Explained by Traditional Risk Factors. Arthritis Rheum (2001) 44:2737–45. doi: 10.1002/1529-0131(200112)44:12<2737::AID-ART460>3.0.CO;2-# 11762933

[2] Elkan AC Engvall IL Cederholm T Hafstrom I. Rheumatoid Cachexia, Central Obesity and Malnutrition in Patients With Low-Active Rheumatoid Arthritis: Feasibility of Anthropometry, Mini Nutritional Assessment and Body Composition Techniques. Eur J Nutr (2009) 48:315–22. doi: 10.1007/s00394-009-0017-y 19333642

[3] Walsmith J Roubenoff R. Cachexia in Rheumatoid Arthritis. Int J Cardiol (2002) 85:89–99. doi: 10.1016/S0167-5273(02)00237-1 12163213

[4] Pereira SS Alvarez-Leite JI. Low-Grade Inflammation, Obesity, and Diabetes. Curr Obes Rep (2014) 3:422–31. doi: 10.1007/s13679-014-0124-9 26626919

[5] Huh JY Park YJ Ham M Kim JB. Crosstalk Between Adipocytes and Immune Cells in Adipose Tissue Inflammation and Metabolic Dysregulation in Obesity. Mol Cells (2014) 37:365–71. doi: 10.14348/molcells.2014.0074 PMC4044307 24781408

[6] Vieira-Potter VJ. Inflammation and Macrophage Modulation in Adipose Tissues. Cell Microbiol (2014) 16:1484–92. doi: 10.1111/cmi.12336 25073615

[7] Al-Suhaimi E Shehzad A. Leptin, Resistin and Visfatin: The Missing Link Between Endocrine Metabolic Disorders and Immunity. Eur J Med Res (2013) 18:12. doi: 10.1186/2047-783X-18-12 23634778 PMC3655867

[8] Tilg H Moschen AR. Adipocytokines: Mediators Linking Adipose Tissue, Inflammation and Immunity. Nat Rev Immunol (2006) 6:772–83. doi: 10.1038/nri1937 16998510

[9] Ajeganova S Andersson ML Hafström I. Association of Obesity With Worse Disease Severity in Rheumatoid Arthritis as Well as With Comorbidities: A Long-Term Followup From Disease Onset. Arthritis Care Res (2013) 65:78–87. doi: 10.1002/acr.21710 22514159

[10] García-Poma A Segami MI Mora CS Ugarte MF Terrazas H-N Rhor EA. Obesity is Independently Associated With Impaired Quality of Life in Patients With Rheumatoid Arthritis. Clin Rheumatol (2007) 26:1831–5. doi: 10.1007/s10067-007-0583-4 17340047

[11] Gremese E Carletto A Padovan M Atzeni F Raffeiner B Giardina AR. Gruppo Italiano Di Studio Sulle Early Arthritis (GISEA).Obesity and Reduction of the Response Rate to Anti-Tumor Necrosis Factor α in Rheumatoid Arthritis: An Approach to a Personalized Medicine. Arthritis Care Res (Hoboken) (2013) 65:94–100. doi: 10.1002/acr.21768 22730143

[12] Van der Helm-van Mil HM van der Kooij SM Allaart CF Toes RE Huizinga TW. A High Body Mass Index has a Protective Effect on the Amount of Joint Destruction in Small Joints in Early Rheumatoid Arthritis. Ann Rheum Dis (2008) 67:769–74. doi: 10.1136/ard.2007.078832 17965124

[13] Jawaheer D Olsen J Lahiff M Forsberg S Lähteenmäki J da Silveira IG. QUEST-R. Gender, Body Mass Index and Rheumatoid Arthritis Disease Activity: Results From the QUEST-RA Study. Clin Exp Rheumatol (2010) 28:454–61. PMC3012645 20810033

[14] Kremers HM Crowson CS Therneau TM Roger VL Gabriel SE. High Ten-Year Risk of Cardiovascular Disease in Newly Diagnosed Rheumatoid Arthritis Patients: A Population-Based Cohort Study. Arthritis Rheum (2008) 58:2268–74. doi: 10.1002/art.23650 PMC2929699 18668561

[15] Arnett FC Edworthy SM Bloch DA McShane DJ Fries JF Cooper NS. The American Rheumatism Association 1987 Revised Criteria for the Classification of Rheumatoid Arthritis. Arthritis Rheum (1988) 31:315–24. doi: 10.1002/art.1780310302 3358796

[16] Aletaha D Neogi T Silman AJ Funovits J Felson DT Bingham CO 3rd. 2010 Rheumatoid Arthritis Classification Criteria. An American College of Rheumatology/European League Against Rheumatism Collaborative Initiative. Arthritis Rheum (2010) 62:2569–81. doi: 10.1002/art.27584 20872595

[17] Solomon DH Garg R Lu B Todd DJ Mercer E Norton T. Effect of Hydroxychloroquine on Insulin Sensitivity and Lipid Parameters in Rheumatoid Arthritis Patients Without Diabetes Mellitus: A Randomized, Blinded Crossover Trial. Arthritis Care Res (Hoboken) (2014) 66:1246–51. doi: 10.1002/acr.22285 PMC4467535 24470436

[18] Matthews DR Hosker JP Rudenski AS Naylor BA Treacher DF Turner RC. Homeostasis Model Assessment: Insulin Resistance and Beta-Cell Function From Fasting Plasma Glucose and Insulin Concentrations in Man. Diabetologia (1985) 28:412–9. doi: 10.1007/BF00280883 3899825

[19] Guzmán-Ruiz R Tercero-Alcázar C Rabanal-Ruiz Y Díaz-Ruiz A El Bekay R Rangel-Zuñiga OA. Adipose Tissue Depot-Specific Intracellular and Extracellular Cues Contributing to Insulin Resistance in Obese Individuals. FASEB J (2020) 34:7520–39. doi: 10.1096/fj.201902703R PMC7384030 32293066

[20] Pfaffl MW Tichopad A Prgomet C Neuvians TP. Determination of Stable Housekeeping Genes, Differentially Regulated Target Genes and Sample Integrity: BestKeeper—Excel-Based Tool Using Pair-Wise Correlations. Biotechnol Lett (2004) 26:509–15. doi: 10.1023/B:BILE.0000019559.84305.47 15127793

[21] Caplan L Davis LA Bright CM Kerr GS Lazaro DM Khan NA. Bodymass Index and the Rheumatoid Arthritis Swollen Joint Count: An Observational Study. Arthritis Care Res (Hoboken) (2013) 65:101–6. doi: 10.1002/acr.21734 PMC3430738 22623288

[22] Choe JY Bae J Lee H Park S Kim S. Lack Association of Body Mass Index With Disease Activity Composites of Rheumatoid Arthritis in Korean Population: Cross-Sectional Observation. Clin Rheumatol (2014) 33:485–92. doi: 10.1007/s10067-013-2427-8 24202616

[23] Versini M Jeandel PY Rosenthal E Shoenfeld Y. Obesity in Autoimmune Diseases: Not a Passive Bystander. Autoimmun Rev (2014) 13:981–1000. doi: 10.1016/j.autrev.2014.07.001 25092612

[24] Liu D Luo S Li Z. Multifaceted Roles of Adiponectin in Rheumatoid Arthritis. Int Immunopharmacol (2015) 28:1084–90. doi: 10.1016/j.intimp.2015.08.013 26307192

[25] Minamino H Katsushima M Yoshida T Hashimoto M Fujita Y Shirakashi M. Increased Circulating Adiponectin is an Independent Disease Activity Marker in Patients With Rheumatoid Arthritis: A Cross-Sectional Study Using the KURAMA Database. PloS One (2020) 15:e0229998. doi: 10.1371/journal.pone.0229998 32126127 PMC7053773

[26] Gomez R Conde J Scotece M Gomez-Reino JJ Lago F Gualillo O. What’s New in Our Understanding of the Role of Adipokines in Rheumatic Diseases? Nat Rev Rheumatol (2011) 7:528–36. doi: 10.1038/nrrheum.2011.107 21808287

[27] Sharma A Kumar A Jha A Agarwal A Misra A. The Impact of Obesity on Inflammatory Markers Used in the Assessment of Disease Activity in Rheumatoid Arthritis - a Cross-Sectional Study. Reumatologia (2020) 58:9–14. doi: 10.5114/reum.2020.93506 32322118 PMC7174796

[28] George MD Giles JT Katz PP England BR Mikuls TR Michaud K. Impact of Obesity and Adiposity on Inflammatory Markers in Patients With Rheumatoid Arthritis. Arthritis Care Res (Hoboken) (2017) 69:1789–98. doi: 10.1002/acr.23229 PMC5634905 28393498

[29] Kapoor D Clarke S Stanworth R Channer KS Jones TH. The Effect of Testosterone Replacement Therapy on Adipocytokines and C-Reactive Protein in Hypogonadal Men With Type 2 Diabetes. Eur J Endocrinol (2007) 156:595–602. doi: 10.1530/EJE-06-0737 17468196

[30] Arias de la Rosa I Escudero-Contreras A Rodríguez-Cuenca S Ruiz-Ponce M Jiménez-Gómez Y Ruiz-Limón P. Defective Glucose and Lipid Metabolism in Rheumatoid Arthritis is Determined by Chronic Inflammation in Metabolic Tissues. J Intern Med (2018) 284:61–77. doi: 10.1111/joim.12743 29532531

[31] Jhun JY Yoon BY Park MK Oh HJ Byun JK Lee SY. Obesity Aggravates the Joint Inflammation in a Collagen-Induced Arthritis Model Through Deviation to Th17 Differentiation. Exp Mol Med (2012) 44:424. doi: 10.3858/emm.2012.44.7.047 22513335 PMC3406287

[32] Kim SJ Chen Z Essani AB Elshabrawy HA Volin MV Fantuzzi G. Differential Impact of Obesity on the Pathogenesis of RA or Preclinical Models is Contingent on the Disease Status. Ann Rheum Dis (2017) 76:731–39. doi: 10.1136/annrheumdis-2016-209206 PMC10026536 27797749

[33] Zhang HH Halbleib M Ahmad F Manganiello VC Greenberg AS. Tumor Necrosis Factor-Alpha Stimulates Lipolysis in Differentiated Human Adipocytes Through Activation of Extracellular Signal-Related Kinase and Elevation of Intracellular cAMP. Diabetes (2002) 5:2929–35. doi: 10.2337/diabetes.51.10.2929 12351429

[34] Giles JT Ferrante AW Broderick R Zartoshti A Rose J Downer K. Adipose Tissue Macrophages in Rheumatoid Arthritis: Prevalence, Disease-Related Indicators, and Associations With Cardiometabolic Risk Factors. Arthritis Care Res (Hoboken) (2018) 70:175–84. doi: 10.1002/acr.23253 28388816

[35] Lee J. Adipose Tissue Macrophages in the Development of Obesity-Induced Inflammation, Insulin Resistance and Type 2 Diabetes. Arch Pharm Res (2013) 36:208–22. doi: 10.1007/s12272-013-0023-8 PMC4086483 23397293

[36] Van Raemdonck K Umar S Szekanecz Z Zomorrodi RK Shahrara S. Impact of Obesity on Autoimmune Arthritis and its Cardiovascular Complications. Autoimmun Rev (2018) 17:821–35. doi: 10.1016/j.autrev.2018.02.007 PMC9996646 29885537

[37] Aletaha D Neogi T Silman AJ Funovits J Felson DT Bingham CO 3rd. 2010 Rheumatoid Arthritis Classification Criteria. An American College of Rheumatology/European League Against Rheumatism Collaborative Initiative. Ann Rheum Dis (2010) 69:1580–8. doi: 10.1136/ard.2010.138461 20699241

[38] Smolen JS Landewé RBM Bijlsma JWJ Burmester GR Dougados M Kerschbaumer A. EULAR Recommendations for the Management of Rheumatoid Arthritis With Synthetic and Biological Disease-Modifying Antirheumatic Drugs: 2019 Update. Ann Rheum Dis (2020) 79:685–99. doi: 10.1136/annrheumdis-2019-216655 31969328

[39] Abdel-Hamid AAM Firgany AL. Favorable Outcomes of Hydroxychloroquine in Insulin Resistance may be Accomplished by Adjustment of the Endothelial Dysfunction as Well as the Skewed Balance of Adipokines. Acta Histochemica (2016) 118:560–73. doi: 10.1016/j.acthis.2016.06.002 27320898

[40] Hage MP Al-Badri MR Azar ST. A Favorable Effect of Hydroxychloroquine on Glucose and Lipid Metabolism Beyond its Anti-Inflammatory Role. Ther Adv Endocrinol Metab (2014) 5:77–85. doi: 10.1177/2042018814547204 25343023 PMC4206615

[41] Rempenault C Combe B Barnetche T Gaujoux-Viala C Lukas C Morel J. Metabolic and Cardiovascular Benefits of Hydroxychloroquine in Patients With Rheumatoid Arthritis: A Systematic Review and Meta-Analysis. Ann Rheum Dis (2018) 77:98–103. doi: 10.1136/annrheumdis-2017-211836 28970215

[42] Mercer E Rekedal L Garg R Lu B Massarotti EM Solomon DH. Hydroxychloroquine Improves Insulin Sensitivity in Obese non-Diabetic Individuals. Arthritis Res Ther (2012) 14:R135. doi: 10.1186/ar3868 22676348 PMC3446518

[43] Nicolau J Lequerré T Bacquet H Vittecoq O. Rheumatoid Arthritis, Insulin Resistance, and Diabetes. Joint Bone Spine (2017) 84:411–6. doi: 10.1016/j.jbspin.2016.09.001 27777170

[44] Chen J Sun J Doscas ME Ye J Williamson AJ Li Y. Control of Hyperglycemia in Male Mice by Leflunomide: Mechanisms of Action. Endocrinol (2018) 237:43–58. doi: 10.1530/JOE-17-0536 PMC5839151 29496905

[45] Mencarelli A Francisci D Renga B D'Amore C Cipriani S Basile F. Ritonavir-Induced Lipoatrophy and Dyslipidaemia is Reversed by the Anti-Inflammatory Drug Leflunomide in a PPAR-γ-Dependent Manner. Antivir Ther (2012) 17:669–78. doi: 10.3851/IMP2039 22297608

